# Inflammatory and cytotoxic effects of acrolein, nicotine, acetylaldehyde and cigarette smoke extract on human nasal epithelial cells

**DOI:** 10.1186/1471-2466-14-32

**Published:** 2014-03-01

**Authors:** David M Comer, Joseph Stuart Elborn, Madeleine Ennis

**Affiliations:** 1 Centre for Infection and Immunity, Health Sciences Building, School of Medicine, Dentistry and Biomedical Sciences, Queen’s University of Belfast, 97 Lisburn Road, Belfast BT9 7BL, UK; 2 Respiratory Department, Belfast City Hospital, Lisburn Road, Belfast BT9 7AB, UK

**Keywords:** Nasal epithelium, Cigarette smoke extract, Acrolein, Nicotine, Acetylaldehyde, Apoptosis

## Abstract

**Background:**

Cigarette smoke induces a pro-inflammatory response in airway epithelial cells but it is not clear which of the various chemicals contained within cigarette smoke (CS) should be regarded as predominantly responsible for these effects. We hypothesised that acrolein, nicotine and acetylaldehyde, important chemicals contained within volatile cigarette smoke in terms of inducing inflammation and causing addiction, have immunomodulatory effects in primary nasal epithelial cell cultures (PNECs).

**Methods:**

PNECs from 19 healthy subjects were grown in submerged cultures and were incubated with acrolein, nicotine or acetylaldehyde prior to stimulation with *Pseudomonas aeruginosa* lipopolysaccharide (PA LPS). Experiments were repeated using cigarette smoke extract (CSE) for comparison. IL-8 was measured by ELISA, activation of NF-κB by ELISA and Western blotting, and caspase-3 activity by Western blotting. Apoptosis was evaluated using Annexin-V staining and the terminal transferase-mediated dUTP nick end-labeling (TUNEL) method.

**Results:**

CSE was pro-inflammatory after a 24 h exposure and 42% of cells were apoptotic or necrotic after this exposure time. Acrolein was pro-inflammatory for the PNEC cultures (30 *μ*M exposure for 4 h inducing a 2.0 fold increase in IL-8 release) and also increased IL-8 release after stimulation with PA LPS. In contrast, nicotine had anti-inflammatory properties (0.6 fold IL-8 release after 50 *μ*M exposure to nicotine for 24 h), and acetylaldehyde was without effect. Acrolein and nicotine had cellular stimulatory and anti-inflammatory effects respectively, as determined by NF-κB activation. Both chemicals increased levels of cleaved caspase 3 and induced cell death.

**Conclusions:**

Acrolein is pro-inflammatory and nicotine anti-inflammatory in PNEC cultures. CSE induces cell death predominantly by apoptotic mechanisms.

## Background

Nasal and sinus inflammation is common in chronic obstructive pulmonary disease (COPD) and contributes to the decline in lung function [1]. The responses of the bronchial epithelium to cigarette smoke (CS) have been well characterised [2], but effects on the nasal epithelium, also important in respiratory disease, are not as well understood. Sinonasal symptoms in COPD range from 75% [3] to as much as 88% [4]. The most commonly reported upper airway symptoms are rhinorrhea, nasal obstruction and sneezing [3,5]. Overall, these observations confirm that significant sinonasal inflammation is present and important in COPD and in smokers [6]. Therefore, dissecting the effects of cigarette smoke on nasal epithelial cells is important in terms of our understanding of inflammation and COPD.

Although CSE is widely used in cell culture research it is an imperfect tool, having been criticised as a poor substitute for the prolonged, chronic exposure of tobacco smoke to which many smokers are exposed [7]. Recently investigators have opted to study individual components of CS [8-10]. The inherent complexity of CSE makes it difficult to identify any individual chemical mediating a particular cellular effect of interest. Furthermore, it is difficult to understand the relevance of any particular CSE concentration and duration of exposure. Even if inferences are made on the basis of findings from CSE data, these experiments do not duplicate in an entirely satisfactory manner all of the components that exist *in vivo*.

CSE has been reported to decrease basal IL-8 in alveolar cell lines [11]. However, other groups have demonstrated that CSE can have *stimulatory* properties in alveolar cell lines [11,12] and in primary human macrophages [13] and to *heighten* the release of IL-8 in submerged healthy bronchial epithelial cell cultures after stimulation [14]. These discrepant findings in alveolar cell lines are almost certainly a result of the different concentrations of CSE used, with lower concentrations stimulating cells and higher concentrations being anti-inflammatory. It is however much more difficult to provide a valid explanation for the inconsistent findings in the primary cell research. Although there is no consensus in relation to the propensity for tobacco smoke to induce a pro-inflammatory response in bronchial epithelial cells, the weight of evidence, on balance, would support a pro-inflammatory [2,15-17] as opposed to an anti-inflammatory effect [18,19].

Despite the diverse chemicals contained within cigarette smoke, its acute effects on cell function and toxicity appear to be due largely to volatile thiol-reactive components, of which acrolein is most abundant and reactive [20]. A toxicological risk assessment of the chemical constituents of cigarette smoke indicated that acrolein and acetylaldehyde had the highest overall non-cancer risk index for respiratory disease [21]. *In vivo* studies demonstrate that acrolein may be responsible for many of the respiratory responses to cigarette smoke exposure. For example, an acute exposure to acrolein diminishes pulmonary defence against bacterial and viral infection in animals [22,23], and a chronic exposure induces bronchial lesions and mucous hyperplasia [24]. Nicotine, a small molecule organic alkaloid, is another important constituent in cigarette smoke which not only is strongly related to addiction [25], but also is at least partially responsible for the airway irritation and inflammation induced by whole CS [26,27]. Acetylaldehyde, in concert with nicotine, contributes to addiction [28]. Furthermore, acetylaldehyde impairs mucociliary clearance in the lung, leading to an impaired host defense [29]. Therefore, acrolein, nicotine and acetylaldehyde are among the most important and relevant chemicals in cigarette smoke.

In addition to the activity of inflammatory cells in the airway, heightened proteolytic activity and greater levels of oxidative stress, an imbalance between apoptosis and proliferation of structural cells in the lung probably contributes to the pathogenesis of COPD [30]. The mechanism of cell death due to CSE exposure remains controversial [31,32]. In these experiments, we hypothesized that acrolein, nicotine and acetylaldehyde, known to be contained within CS, individually immunomodulate primary nasal epithelial cells (PNEC) cultures. CSE has been shown to be cytotoxic to nasal epithelial cell cultures [33], and we aimed to establish which, if any, of these individual chemicals were contributory.

## Methods

### Study subjects and ethics statement

Nasal brushings were obtained from 19 healthy volunteers. All 19 subjects were non-smokers, nor did any have chronic respiratory symptoms or require any therapy used for respiratory diseases. Bilateral nasal brushings were performed using a bronchial cytology brush (TeleMed Systems Inc., MA, USA) from the medial aspect of the inferior turbinate as previously described [34]. The brush was then removed and rinsed thoroughly in a 15 ml polypropylene tube containing sterile PBS. Provided the patient tolerated the procedure, two brushings were obtained from each nostril. At the end of the procedure, DMEM medium containing 10% Fetal Bovine Serum, Penicillin Streptomycin antibiotics (Invitrogen, USA) and Primocin (Invivogen, USA) was added. This study was approved by the Office for Research Ethics Committees Northern Ireland (REC: 09/NIR03/42) and all participants provided written informed consent.

### Cell culture and soluble mediator release

**Figure 1:**
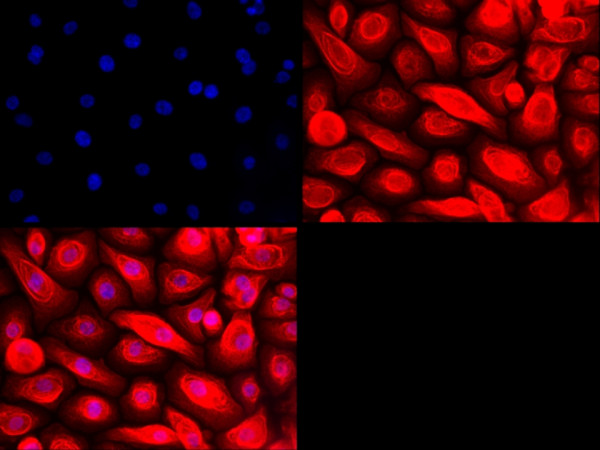
**Immunocytochemistry for cytokeratin 5 in nasal epithelial cell cultures.** Primary nasal epithelial cell cultures were stained with a rabbit anti-human antibody against cytokeratin 5 (1:100). The primary antibody was detected using a secondary antibody coupled to Alexafluor 568 (1:500). Nuclei were stained blue with DAPI (×40). Each quadrant represents DAPI staining alone (upper left), staining for cytokeratin 5 alone (upper right), and staining for both DAPI and cytokeratin 5 (lower left).

PNECs were expanded in bronchial epithelial growth medium (BEGM, Promocell) with 100 units/ml Penicillin Streptomycin antibiotics (Invitrogen, USA) and 100 *μ*g/ml Primocin (Invivogen, USA). Cells were confirmed to be epithelial in origin by randomly staining cultures by immunocytochemical staining for cytokeratin expression (Figure 1). Cells (passage 2-3; 1 × 10^5^ cells/ml) were seeded in purified bovine collagen coated (PureCol; Advanced Biomatrix) 12-well plates and cultured for 24 h. Cells were stimulated with LPS from *Pseudomonas aeruginosa* (Sigma-Aldrich), either with or without pretreatment with acrolein, nicotine or acetylaldehyde as outlined in the results section. Separate cultures were treated with 5% CSE alone. After appropriate stimulation, cell-free supernatants were collected and stored at -80° for future IL-8 and IL-6 measurement using the Duoset ELISA kits (R&D Systems) according to the manufacturer’s instructions. Cells were used for protein expression studies.

### Immunofluorescence

Cells were seeded on coverslips at a density of 1 × 10^5^ cells/ml. The following day, cells were fixed in 4% PFA and washed in PBS. After permeabilisation with 0.2% Triton-X 100 (Sigma, Dorset, UK), cells were treated with a 1:100 dilution of rabbit anti-cytokeratin-5 primary antibody (Abcam, Cambridge, UK) overnight at 4°C. The primary antibody was detected using a 1:500 dilution of Alexafluor 568 goat anti-rabbit IgG (Invitrogen Ltd., Paisley, UK). Nuclei were stained with DAPI (Vector Laboratories, Peterborough, UK). Images were captured using LAS AF (Leica) acquisition software.

### CSE preparation

CSE was prepared by a modification of the method of Richter *et al.*[17] One commercial Marlboro Red cigarette (0.8 mg nicotine; 10 mg Tar; 10 mg carbon monoxide) was combusted with a modified syringe-driven apparatus. The smoke was bubbled through 25 ml of media over 5 min by drawing 35-ml volume of smoke every 15 s. The resulting suspension was filtered through a 0.2 *μ*m pore-size filter to remove large particles and bacteria. This solution was regarded as “100% CSE” and freshly generated for each experiment, and subsequently serially diluted with culture medium to obtain a final 5% working concentration. The optical density was constant when comparing a series of 5% CSE solutions prepared in this manner [35].

### Annexin-PI staining

Apoptosis was analysed using Annexin V (Av) and Propidium Iodide (PI) staining (eBioscience, UK). Events which were negative for Av and PI were considered to represent viable cells, those positive for Av, but negative for PI represented early apoptosis and events positive for both Av and PI secondary necrosis. To eliminate debris from the analysis the discrimination level was set to 100. Cells, adjusted to 1 × 10^5^ cells per 100 *μ*l, were suspended in binding buffer and incubated with the fluorochrome-conjugated Av for 15 min. Subsequently, after washing, cells were re-suspended in fresh binding buffer and stained with PI. In order to set gates and establish appropriate compensation settings, cells were stained with PI alone, Av alone, and both PI and Av. In order to obtain positive control samples for early apoptosis and necrosis respectively, cells were treated with 5 *μ*M staurosporin and 0.1% Triton-X respectively for 4 h.

### TUNEL assay

After treating PNECs with individual chemicals as described in the results section, cultures were analysed for apoptosis using the Click-It TUNEL assay (Invitrogen, UK). Cells, after being fixed and permeabilised, were exposed to a reaction cocktail overnight at room temperature. Cells were then treated with a reaction buffer additive mixture for 30 min, and cells mounted onto glass coverslips with mounting media and DAPI. Images were captured using LAS AF (Leica) acquisition software.

### Western blotting

For Western blotting, cells were lysed in radioimmunoprecipitation assay buffer (Sigma-Aldrich, UK). The protein concentration was determined by BCA protein assay kit (Fisher Scientific, UK). Equal amounts of protein (15 *μ*g/lane) were separated in a 10% SDS gel, and transferred to a polyvinylidene difluoride sheet by electroelution with a constant voltage of 100 V for 90 min at room temperature. After blocking with 0.1% Tween 20 supplemented with PBS (T-PBS) containing skimmed milk, the sheet was incubated with a 1:1,000 dilution phosho-NF-κB antibody (Cell Signalling, UK) at 4°C overnight. The sheet was then washed three times with T-PBS, and incubated with goat HRP conjugated anti-rabbit antibody (1:3,000 dilution; Abcam, UK) for 1 h at room temperature. After washing with T-PBS three times, immunoreactive protein bands were revealed with an enhanced chemiluminescence western blot analysis system. After being “stripped” using a Western Blot Stripping Buffer (Thermoscientific, UK), membranes were re-probed with a polyclonal antibody against IκB-α and for beta-actin (both 1:1,000 dilution).

To determine the effects of individual chemicals on caspase-3 activation in PNEC cultures, cultures were treated as described in the results section. For the detection of cleaved caspase-3, a 1:1,000 dilution of primary antibody was used (Cell Signalling, UK). The sheet was then washed three times with T-PBS, and incubated with goat HRP conjugated anti-rabbit antibody (1:3,000 dilution; Abcam, UK) for 1 h at room temperature and developed as described.

### TransAM NF-κB assay

Nuclear extracts were prepared using a nuclear extraction kit from Active Motif (Belgium, UK) according to manufacturer’s instructions. The Active Motif Trans-AM NF-κB ELISA kit (Belgium, UK) was used to determine the levels of p65 in nuclear extracts. In brief, 2 *μ*g of nuclear extract, diluted to 20 *μ*L, was added to the wells coated with oligonucleotides containing the NF-κB consensus binding site. The primary antibodies used to detect NF-κB recognise an epitope on p65 that is accessible only when NF-κB is activated and bound to its target DNA. After the addition of secondary antibodies conjugated with HRP and substrate, absorbance was read at 450 nm (with a reference wavelength at 650 nm). In order to monitor for specificity, competitive binding assays were performed. Wild-type or mutated consensus oligonucleotides were added to the wells containing immobilised oligonucleotides before the addition of nuclear extracts.

### Statistics

Statistical analysis was performed using SPSS version 17.0 (SPSS inc., Chicago, IL, USA). Data are presented as median values ± interquartile range. Comparisons between groups were performed using the nonparametric Kruskal-Wallis test for multiple comparisons and the Mann–Whitney test for two groups. A *p* value of less than 0.05 was considered significant.

## Results

### Soluble mediator release

**Figure 2:**
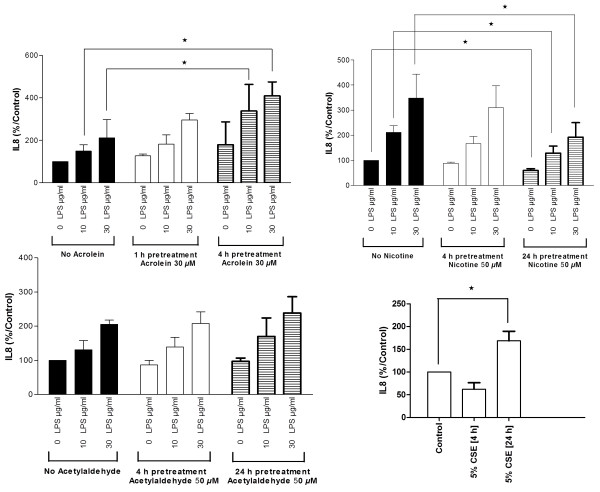
**IL-8 release from PNECs after 24 h treatment with PA LPS in the presence or absence of 30** ***μ*M acrolein for 1 h or 4 h, 50** ***μ*M nicotine or 50** ***μ*M acetylaldehyde for 4 h or 24 h, and 5% CSE for 4 h or 24 h.** PNEC cultures were treated with increasing concentrations of LPS for 24 h ± pretreatment with acrolein, nicotine or acetylaldehyde (n = 4). Separate cultures were treated with 5% CSE for 4 h or 24 h (n = 4). Data are displayed as median ± IQR. * *p* < 0.05. The control IL-8 concentration was 1310 ± 223 pg/ml.

Cells were confirmed to be epithelial by staining with cytokeratin-5 (Figure 1). Constitutive and stimulated release of IL-8 and IL-6 was used to determine PNEC activation. Pre-treating PNEC cultures with 30 *μ*M acrolein for 1 h or 4 h increased the basal constitutive release of IL-8, and heightened the release of IL-8 after subsequent stimulation with *Pseudomonas aeruginosa* LPS (PA LPS; Sigma-Aldrich, UK). This was statistically significant after a 4 h incubation period with acrolein (Figure 2). Repeating experiments using 50 *μ*M nicotine (Sigma-Aldrich, UK) demonstrated anti-inflammatory effects for the PNEC cultures after a 4 h and 24 h incubation period, although only reached statistical significance for the latter incubation period. 50 *μ*M acetylaldehyde (Sigma-Aldrich, UK) was without effect on cytokine release after a 4 h and 24 h incubation period. Treating PNEC cultures with CSE for 24 h heightened the release of IL-8 (Figure 2). In separate experiments, PNEC cultures were treated for 4 h with acrolein (10–50 *μ*M) which demonstrated a dose responsive increment in IL-8 release up to 30 *μ*M with evidence of cytotoxicity at higher concentrations determined using the MTT assay. Results for IL-6 followed a similar trend for all experiments (data not shown).

### Effects of acrolein, nicotine, acetylaldehyde and CSE on activation of caspase-3 in PNEC cultures

**Figure 3:**
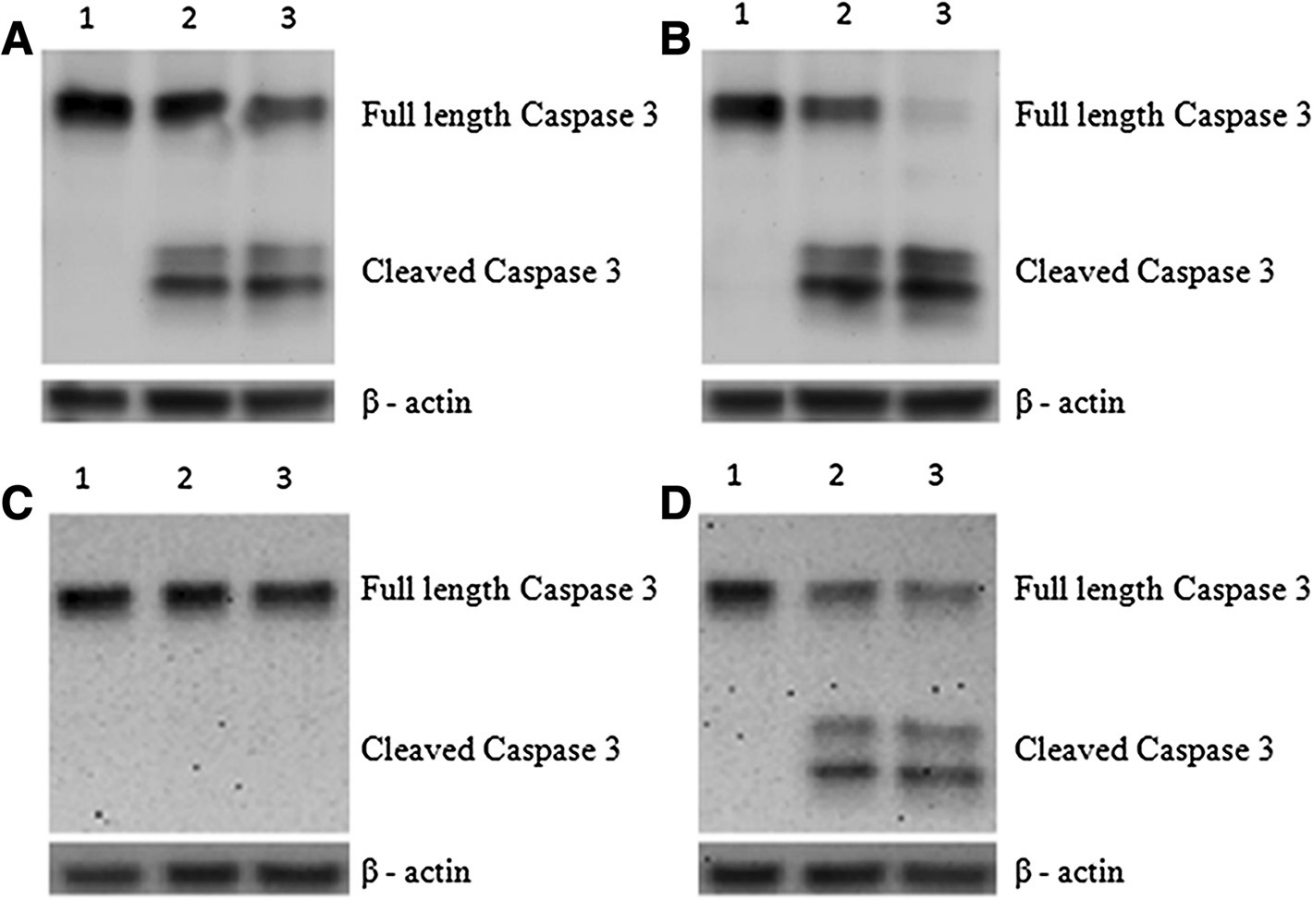
**Effect of acrolein and nicotine on caspase-3 activation in PNEC cultures determined using western blotting.** Western blots of cleaved caspase 3 in healthy PNECs (with beta-actin loading controls). Blot **A**: lanes 1–3 represent treatment of PNECs with 0, 5, 50 *μ*M nicotine (1 h); blot **B**: lanes 1–3: 0, 10, 50 *μ*M acrolein (1 h); blot **C**: lanes 1–3 represent treatment of PNECs with 0, 5, 50 *μ*M acetylaldehyde (1 h) and blot **D** lanes 1–3: 0%, 5%, 50% CSE (1 h) respectively.

Using an antibody against the cleaved fragment of caspase 3 (17–19 kDa), we demonstrated by Western blotting that treatment with both acrolein and nicotine for 1 h increased levels of active caspase 3, which was most pronounced for acrolein. Repeating experiments with acetylaldehyde for 1 h did not activate caspase 3. A brief exposure for 1 h to 5% CSE cleaved caspase 3, in a concentration dependant manner (Figure 3).

### Effects of acrolein, nicotine, acetylaldehyde and CSE on cell viability in PNEC cultures

**Figure 4:**
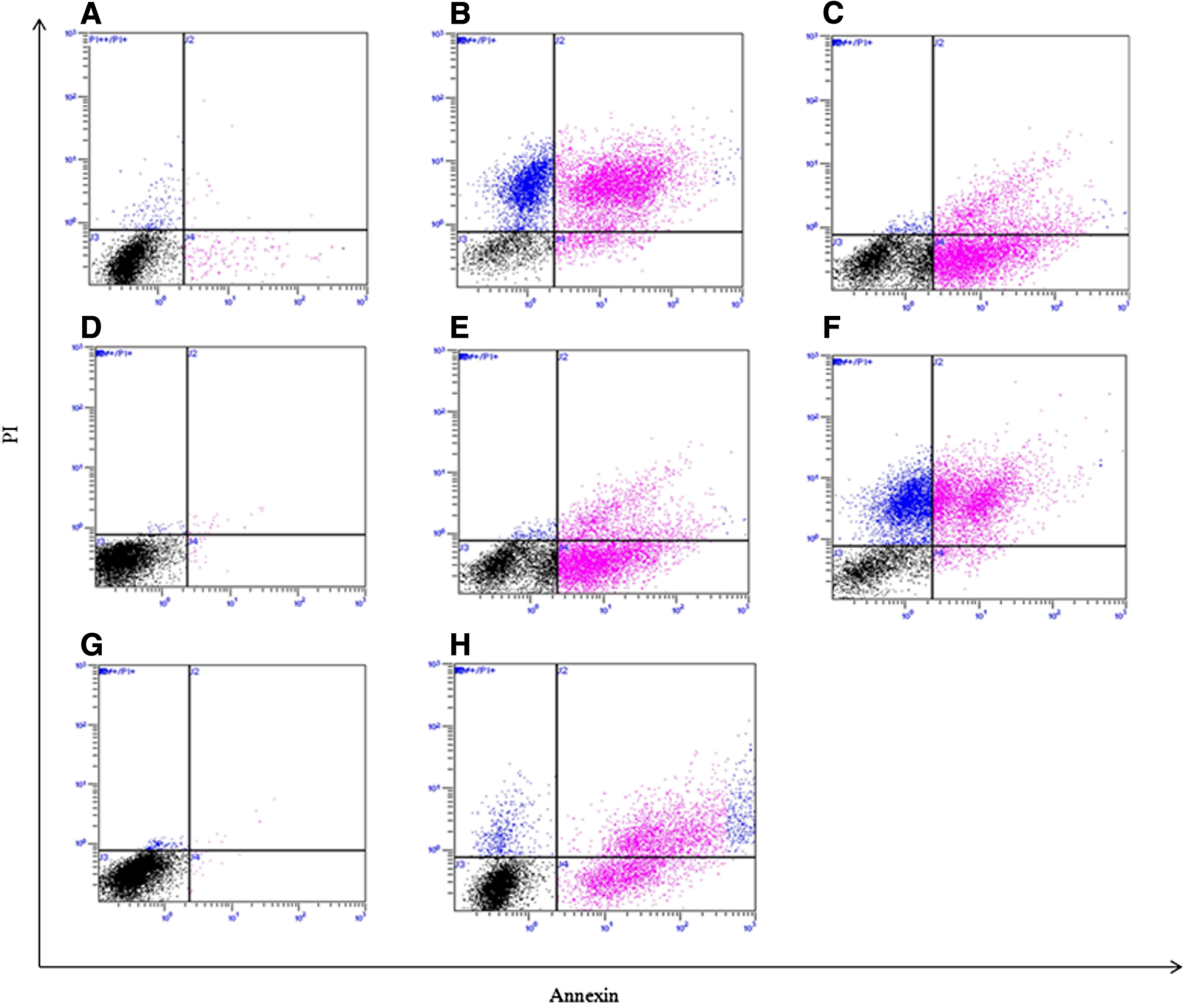
**Effect of acrolein, nicotine, acetylaldehyde and CSE treatment on cell viability.** In each plot, the horizontal axis represents intensity of staining for Annexin V and vertical axis intensity of staining for PI (determined in the FL1 and FL3 plot respectively, both logarithmic scale). Dot plots represent **(A)** untreated cells, **(B)** cells treated with Triton-X for 4 h, **(C)** cells treated with 5 *μ*M staurosporin for 4 h, **(D)** cells treated with media alone for 4 h, **(E)** cells treated with 50 *μ*M nicotine for 24 h, **(F)** cells treated with 50 *μ*M acrolein for 4 h, **(G)** cells treated with 50 *μ*M acetylaldehyde for 24 h, and **(H)** cells treated with 5% CSE for 24 h.

**Figure 5:**
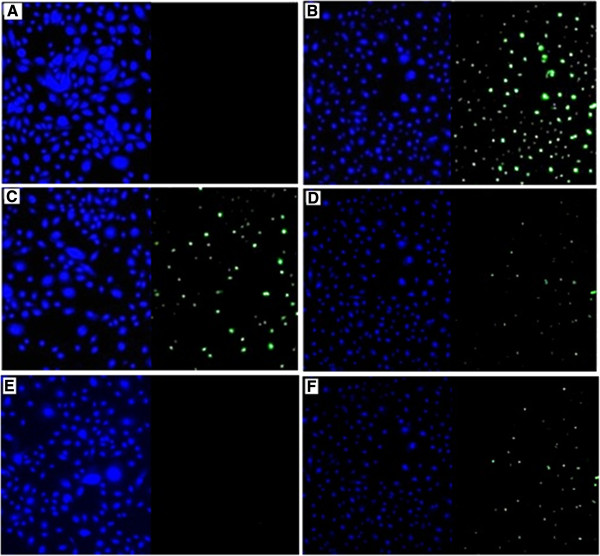
**Effect of acrolein, nicotine, acetylaldehyde and CSE on apoptosis in PNEC cultures using TUNEL assay.** Nasal epithelial cells were grown on coverslips and treated with **(A)** PBS or **(B)** DNase I solution for the negative and positive control respectively. Separate samples were treated with **(C)** 50 *μ*M acrolein for 4 h, **(D)** 50 *μ*M nicotine for 24 h, **(E)** 50 *μ*M acetylaldehyde for 24 h or **(F)** 5% CSE for 24 h. Levels of apoptosis were determined using the Click-iT reaction according to manufacturer’s instructions.

Annexin-V staining demonstrated that 50 *μ*M nicotine exposure for 24 h induced predominantly early apoptosis in PNEC cultures. After treatment with 50 *μ*M acrolein for 4 h, there was evidence of predominantly necrosis. Acetylaldehyde had minimal effects on cell viability, and 5% CSE for 24 h induced apoptosis and secondary necrosis. Events which were positive for apoptosis and necrosis are shown (Figure 4 and Table 1). Apoptosis was confirmed using the TUNEL assay for each stimulus (Figure 5).

**Table 1:** Annexin-V/Propidium iodide analysis of acrolein, nicotine, acetylaldehyde and CSE treatment in PNEC cultures

	**Av (-) PI (-)**	**Av (+) PI (-)**	**Av (+) PI (+)**	**Av (-) PI (+)**
Media 24 h	99.1%	0.4%	0.4%	0.1%
Nicotine 24 h	39.7%	45.5%	13.0%	1.8%
Acrolein 4 h	26.3%	2.2%	39.8%	31.7%
Acetylaldehye 24 h	97.4%	0.2%	2.0%	0.4%
5% CSE 24 h	51.8%	19.1%	25.6%	3.5%

Percentages of events positive for apoptosis and necrosis are shown corresponding to dot-plots D-H in Figure 4.

### Effects of acrolein, nicotine, acetylaldehyde and CSE on NF-κB activation

**Figure 6:**
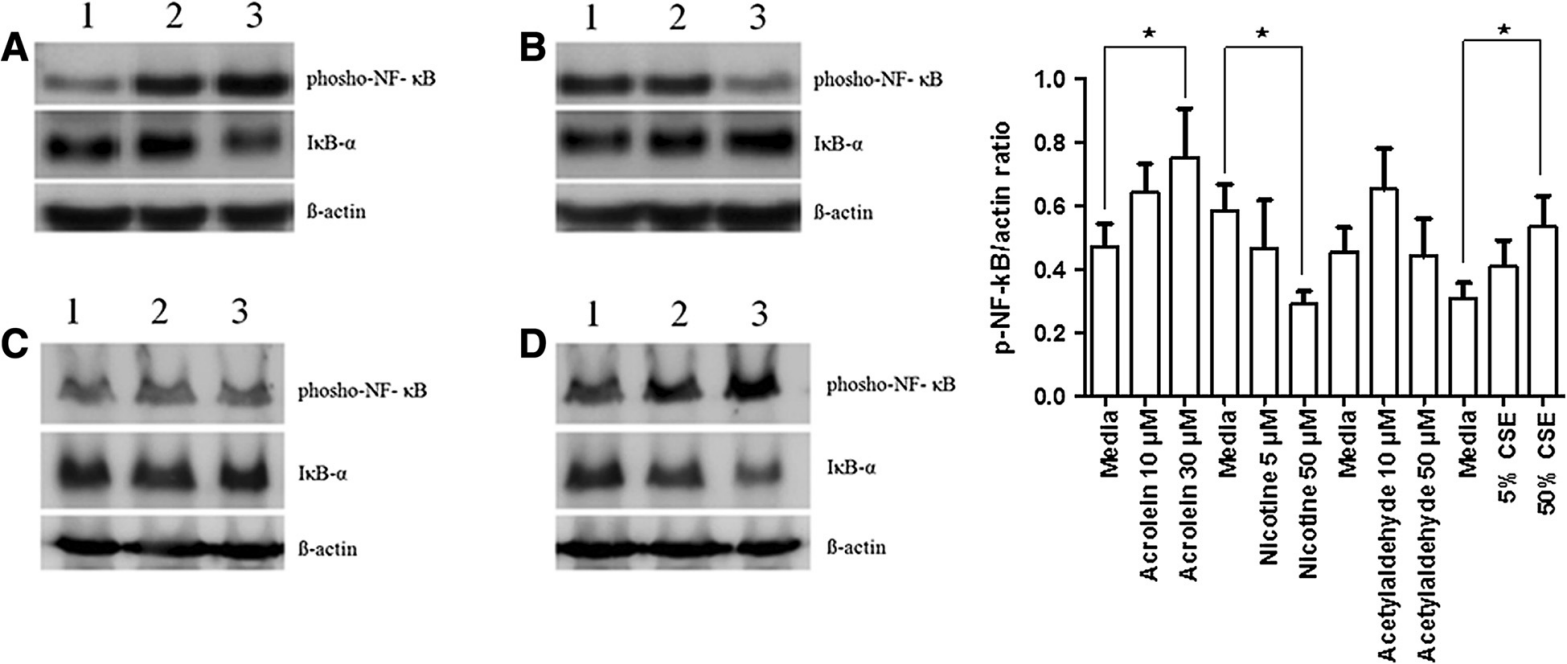
**Effect of acrolein, nicotine and acetylaldehyde on NF-κB expression in PNEC cultures determined using western blotting.** Western blot of phosho-NF-κB and IκB-α protein expression in PNECs (with beta-actin loading controls). Blot **A**: lanes 1–3 represent treatment of PNECs with 0, 10, 30 *μ*M acrolein (1 h); blot **B**: lanes 1–3: 0, 5, 50 *μ*M nicotine (1 h); blot **C**: lanes 1–3 represent treatment of PNECs with 0, 10, 50 *μ*M acetylaldehyde (1 h) and blot **D** lanes 1–3: media, 5% CSE, 50% CSE (1 h). Densitometry included for phosho-NF-κB: actin ratio (n = 5 for each group). Data are displayed as median ± IQR. *p < 0.05.

**Figure 7:**
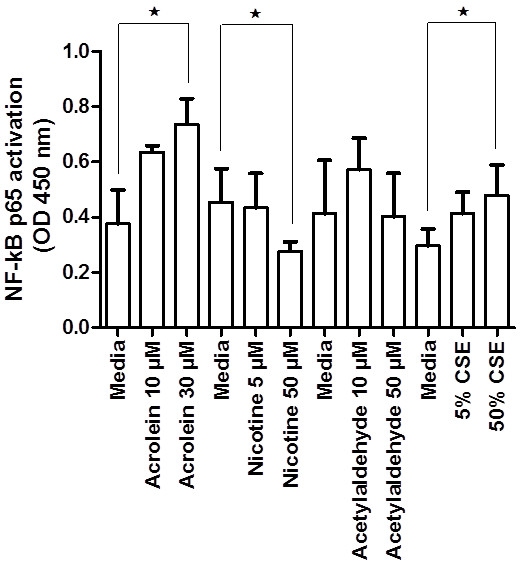
**Effect of acrolein, nicotine and acetylaldehyde on NF-κB p65 activation in PNEC cultures determined using the TransAM NF-κB p65 kit.** Nuclear extracts from PNEC cultures after treatment with 0, 10, 30 *μ*M acrolein (1 h), 0, 5, 50 *μ*M nicotine (1 h), 0, 10, 50 *μ*M acetylaldehyde (1 h) or media, 5% CSE or 50% CSE (1 h) were assayed for NF-κB p65 activation using the TransAM NF-κB p65 Kit (n = 5). Data are displayed as median ± IQR. *p < 0.05.

There was a significant increase in phosho-NF-κB protein levels after stimulation with 30 *μ*g/ml acrolein for 1 h, whereas 50 *μ*M nicotine for 1 h reduced phosho-NF-κB protein concentration measured from whole cell lysates. Repeating experiments using 50 *μ*M acetylaldehyde had no effect on levels of phosho-NF-κB. 50% CSE heightened phosho-NF-κB after 1 h exposure. Levels of IκB-α had an inverse relationship to phosho-NF-κB for all chemical stimulation (Figure 6). Repeating experiments and determining levels of active NF-κB p65 protein from nuclear extracts provided similar results (Figure 7).

## Discussion

Acrolein is pro-inflammatory and nicotine anti-inflammatory in PNEC cultures. In contrast, acetylaldehyde was without effect on cytokine release. Nicotine induced predominantly early apoptosis in the cultures, whereas acrolein induced both apoptosis and overt necrosis. Interestingly, a study exposing PNECs to CSE for 1 h, 2 h, and 4 h reported a time and dose-dependent cytotoxicity of CSE [33]. Nicotine and acrolein increased levels of active caspase 3; acrolein to a greater extent. Experiments were repeated with CSE, containing all of these chemicals, for comparison purposes. We exposed PNEC cultures to CSE for a 4 h and a 24 h period to facilitate comparison of our data with other published work. The concentrations of CSE used can range from 100% CSE for 15 minutes [18] to a 1% CSE for 24 hours (in those studies which use a single cigarette to prepare the initial “100%” stock CSE) [12]. CSE also cleaved full length caspase 3, induced early and late apoptosis after a 24 h exposure, and was pro-inflammatory after this exposure time. Acrolein and CSE increased, whereas nicotine reduced, levels of active NF-κB.

Treating submerged or air liquid interface PNEC cultures with acrolein has been reported to suppress basal IL-8 release [36], and acrolein inhibited LPS-induced cytokine release from human alveolar macrophages when used at a 25 *μ*M concentration [37]. Furthermore, acrolein has been shown to reduce inflammatory responses in human bronchial epithelial cell lines by suppressing NF-κB activation [38]. The report demonstrating anti-inflammatory properties of acrolein in PNEC cultures acquired samples from subjects with chronic rhinosinusitis, and so this may not reflect the activity of acrolein from the PNEC cultures. Furthermore, those reports indicating that acrolein had anti-inflammatory properties on epithelial cells had both treated cultures for 30 minutes [36,38], whereas an 18 h exposure of acrolein in small airway epithelial cell cultures was pro-inflammatory when similar concentrations of acrolein were used [39]. Therefore, and as we have demonstrated in these experiments, it is clear that exposure time is an important determinant as to the effects of acrolein in airway epithelial cell cultures, as is the case for treatment with CSE.

Nicotine had no significant effect on LPS-induced IL-8 production in the CF cell line, CFTE29o [40], but suppressed inflammatory responses in human bronchial epithelial cell lines after stimulation with LPS [41]. Our data indicate that in healthy PNEC cultures, nicotine was anti-inflammatory. In contrast, Tsai *et al.* demonstrated that treatment of bronchial epithelial cell lines with nicotine induced IL-8 production, and this effect was mediated by both ERK and JNK MAPK, but yet not through the p38 pathway [42]. In their study the cell lines were exposed to nicotine for a shorter period of time (4 h, 8 h, 10 h) using a lower concentration (5 *μ*M). In our studies acetylaldehyde appears to have no role in terms of the immunomodulatory effects of CSE. The differences observed by previous investigators are likely to be a result of the different cell types used in their respective experiments.

The concentrations of nicotine and acrolein in the alveolar lining fluid in smokers are of the order of 30 *μ*M and 80 *μ*M respectively [43]. However, maximum stimulation of IL-8 was achieved in primary small airway epithelial cells after stimulation with 30 *μ*M acrolein for 18 h, decreasing thereafter with higher concentrations, attributed to the cytotoxic effects of treatment [39]. Similar results have been reported using the human bronchial cell line, HBE1, with no detectable apoptosis using a flow cytometry method after exposure to 25 *μ*M acrolein for 12 h, but clearly present with higher concentrations [44]. We also report that the optimal acrolein concentration is in the 30 *μ*M range for cell culture experiments, with a marked reduction in cell viability at 50 *μ*M using the MTT assay, rendering the release of IL-8 difficult to interpret at this particular concentration.

Nicotine has been reported to have anti-inflammatory effects in inflammatory bowel disease, and reduces inflammatory cytokine release in alveolar macrophages [45-47]. Previous studies have reported that nicotine exerts its anti-inflammatory function to activation of its receptor, nAChR, and as a consequence, inhibition of the NF-κB pathway [48,49]. Furthermore, nicotine has the potential to inhibit TLR-2 mediated inflammation in response to TLR-2 agonists in CF cell lines [40]. Unfortunately, the use of nicotine as a therapeutic agent is limited by its negative side effects including cardiovascular disease, hypertension, cancer and gastrointestinal disorders [50].

CSE has been reported to induce apoptosis in primary bronchial epithelial cells [51,52], and primary nasal epithelial cells [33], by some but not all investigators [53,54]. Interestingly, one group reported that cigarette smoke condensate (CSC) *inhibited* apoptosis, but yet *caused* necrosis in human umbilical vein endothelial cells [55]. Furthermore, recent research using primary bronchial epithelial cells indicated that CSE caused necrosis rather than apoptosis [56]. This group prepared CSE using 10 ml of media, and so could be considered to be more than twice as concentrated when compared to our CSE preparation. In addition, the epithelial cells were exposed to the CSE for 48 h, and so it is not particularly surprising that no apoptosis was evident as the cells had undergone necrosis. CSC, which also contains the lipid soluble fractions of volatile smoke, induces apoptosis in A549 cells [57]. It is difficult to relate this to other CSE preparations for comparison purposes, or to the *in vivo* airway of the smoker, but it provides evidence that apoptosis can be induced in cell culture with various aqueous CSE preparations. Interestingly, CSE induces cell death in bronchial smooth muscle cells which is blocked by the addition of N-acetylcysteine [45].

The three methods we opted to use in order to determine the loss of cell viability, when considered in isolation, are not ideal. Although the TUNEL nick-end labeling method is increasingly applied to investigate active cell death, many investigators do not regard the technique to be acceptable [55,58]. It has been suggested that DNA fragmentation, which is the fundamental aspect which the TUNEL assay measures, is common to different types of cell death, and so on this basis it cannot itself be relied upon to specifically measure apoptosis [58]. Cell death can occur by mechanisms which are independent of caspase activity [59], and annexin-V staining can give false positives due to damage to the cell membrane induced during the detachment of the cells by trypsin/EDTA. However, despite the shortcomings of the individual assays, and taking all of the evidence together, our data indicate that acrolein, nicotine and CSE are inherently cytotoxic, and that CSE causes cell death by apoptotic mechanisms.

In contrast to our findings, other groups have demonstrated that CSE increased phosphorylated NF-κB, induced DNA damage, yet was protective against apoptosis in BEAS-2B cells [54]. This may be attributable to the fact that cell lines were used and not primary cells, and also likely because a relatively brief 30 minute exposure of CSE was used, a much shorter duration to that which we adopted. Interestingly, acrolein induced apoptosis in the bronchial epithelial cells line, HBE1, which was attributed to the intracellular generation of oxidants [44].

## Conclusion

Our data demonstrate that acrolein and nicotine, when used at concentrations which are of relevance in the airway of cigarette smokers, stimulate and immunosuppress PNEC cultures respectively. Acetylaldehyde, on the other hand, had no effect. Both acrolein and nicotine should be considered toxic substances to cell cultures, and particularly so for acrolein. As acrolein heightened IL-8 release, yet still caused loss of cell viability, this finding at least suggests that the reduced IL-8 release after nicotine treatment cannot merely be attributed to loss of cell viability. This is supported by the reduced levels of phosho-NF-κB apparent after nicotine treatment. Finally, exposure to CSE heightened phosho-NF-κB levels in the PNEC cultures. Overall our data confirm the understanding that CSE is cytotoxic, and that some of the chemicals contained within it can individually immunomodulate and induce cell death in PNEC cultures to varying degrees.

## Abbreviations

CS: Cigarette smoke; CSE: Cigarette smoke extract; CSC: Cigarette smoke condensate; PNECs: Primary nasal epithelial cell cultures; PA LPS: *Pseudomonas aeruginosa* lipopolysaccharide; TUNEL: Terminal transferase-mediated dUTP nick end-labeling; COPD: Chronic obstructive pulmonary disease.

## Competing interests

The authors declare that they have no competing interests.

## Authors’ contributions

JSE, ME and DC conceived of the study, and participated in its design and coordination. DC carried out the laboratory work, recruited study subjects drafted the manuscript. JSE and ME also helped to draft the manuscript. All authors read and approved the final manuscript.

## Pre-publication history

The pre-publication history for this paper can be accessed here:

http://www.biomedcentral.com/1471-2466/14/32/prepub
